# A real or apparent decrease in glomerular filtration rate in patients using olaparib?

**DOI:** 10.1007/s00228-020-03070-0

**Published:** 2020-12-14

**Authors:** M. A. C. Bruin, C. M. Korse, B. van Wijnen, V. M. T. de Jong, S. C. Linn, B. van Triest, H. Rosing, J. H. Beijnen, D. van den Broek, A. D. R. Huitema

**Affiliations:** 1 Department of Pharmacy & Pharmacology, The Netherlands Cancer Institute – Antoni van Leeuwenhoek, Plesmanlaan 121, 1066 CX Amsterdam, The Netherlands; 2 Department of Laboratory Medicine, The Netherlands Cancer Institute, Amsterdam, The Netherlands; 3 Department of Molecular Pathology, The Netherlands Cancer Institute, Amsterdam, The Netherlands; 4 Department of Medical Oncology, The Netherlands Cancer Institute, Amsterdam, The Netherlands; 5 Department of Radiation Oncology, The Netherlands Cancer Institute, Amsterdam, The Netherlands; 6 Department of Clinical Pharmacy, University Medical Center Utrecht, Utrecht University, Utrecht, The Netherlands; 7 Department of Pharmacology, Princess Máxima Center for Pediatric Oncology, Utrecht, The Netherlands

**Keywords:** Olaparib, Creatinine, Cystatin C, eGFR, Renal function

## Abstract

**Purpose:**

Olaparib is a poly (ADP-ribose) polymerase (PARP) inhibitor indicated for ovarian and metastatic breast cancer. Increased serum creatinine levels have been observed in patients taking olaparib, but the underlying mechanism is unknown. This study aimed to investigate if patients receiving olaparib have increased creatinine levels during olaparib treatment and whether this actually relates to a declined glomerular filtration rate (GFR).

**Methods:**

We retrospectively identified patients using olaparib at the Netherlands Cancer Institute – Antoni van Leeuwenhoek (NKI-AVL) from 2012 until 2020. Patients with at least one plasma or serum sample available at baseline/off treatment and during olaparib treatment were included. Cystatin C levels were measured, creatinine levels were available and renal function was determined by calculating the estimated glomerular filtration rate (eGFR) using the Creatinine Equation (CKD-EPI 2009) and the Cystatin C Equation (CKD-EPI 2012).

**Results:**

In total, 66 patients were included. Olaparib treatment was associated with a 14% increase in median creatinine from 72 (inter quartile range (IQR): 22) μmol/L before/off treatment to 82 (IQR: 20) μmol/L during treatment (*p* < 0.001) and a 13% decrease in median creatinine-derived eGFR from 86 (IQR: 26) mL/min/1.73 m^2^ before/off treatment to 75 (IQR: 29) mL/min/1.73 m^2^ during treatment (*p* < 0.001). Olaparib treatment had no significant effect on median cystatin C levels (*p* = 0.520) and the median cystatin C–derived eGFR (*p* = 0.918).

**Conclusions:**

This study demonstrates that olaparib likely causes inhibition of renal transporters leading to a reversible and dose-dependent increase in creatinine and does not affect GFR, since the median cystatin C–derived eGFR was comparable before/off treatment and during treatment of olaparib. Using the creatinine-derived eGFR can give an underestimation of GFR in patients taking olaparib. Therefore, an alternative renal marker such as cystatin C should be used to accurately calculate eGFR in patients taking olaparib.

## Introduction

Olaparib is a potent poly (ADP-ribose) polymerase (PARP) inhibitor approved for the treatment of patients with ovarian cancer and patients with breast cancer (BRCA)–mutated human epidermal growth factor receptor-2 (HER2) negative metastatic breast cancer [1, 2]. Olaparib is orally administrated at a recommended dose of 300 mg (tablets) or 400 mg (capsules) twice a day (BID). In patients taking the recommended dose, increased serum creatinine levels (up to 23%) over baseline were observed [2].

**Fig. 1:**
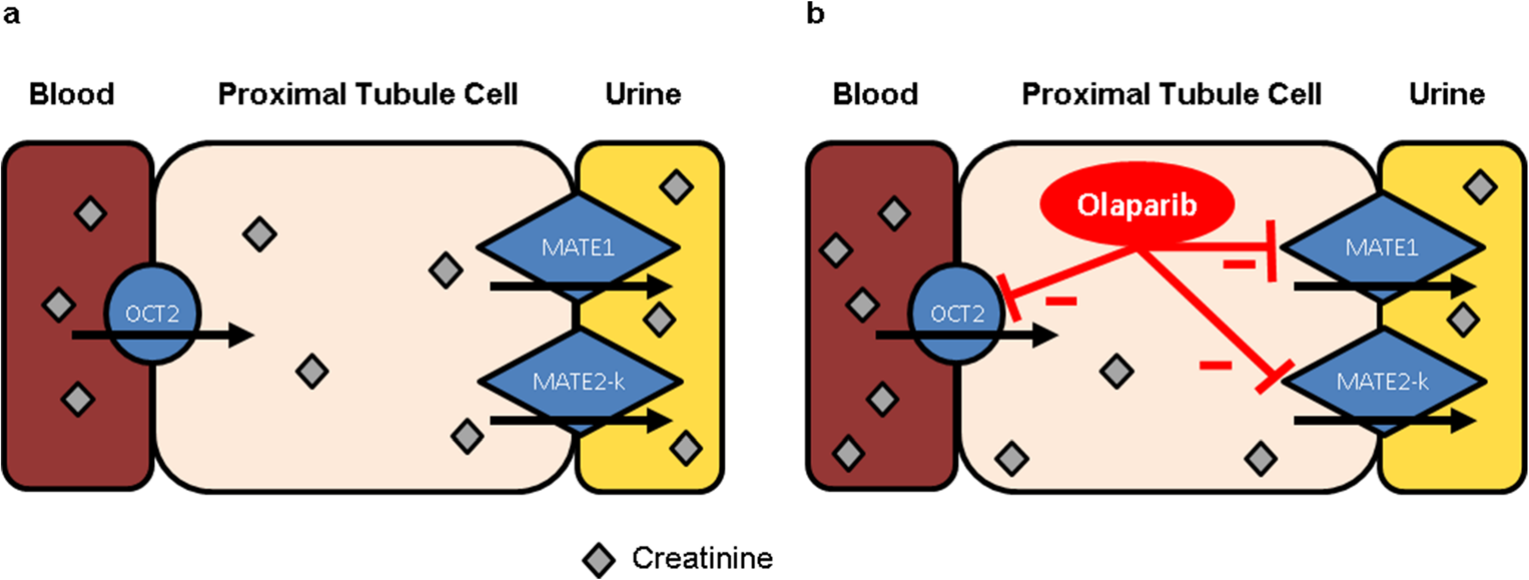
In the kidneys, creatinine is transported by the OCT2 transporter on the basolateral membrane into the proximal tubule cells and subsequently transported to the urine by MATE1- and MATE2-K transporters on the apical membrane (**a**). Inhibition of active transport of creatinine by inhibition of the OCT2, MATE1, and MATE2-K transporters by olaparib could cause an increase in creatinine in blood (**b**)

Creatinine is an endogenous product of creatine metabolism in muscles and is used as a marker to calculate the glomerular filtration rate (GFR). The GFR is used as a functional parameter to evaluate renal function of patients in clinical practice. Creatinine is primarily filtered by the glomerulus, but active tubular secretion accounts for a substantial part of creatinine clearance as well (10–40%) [3]. Active tubular secretion of organic compounds including drugs, toxins, and endogenous compounds is mediated by multiple solute carrier (SLC) transporters in the kidney. Active tubular secretion of creatinine is mainly driven by the organic cation transporter 2 (OCT2) expressed on the basolateral membrane and the multidrug and toxin extrusion protein (MATE) 1 and MATE2-K expressed on the apical membrane of the proximal tubule cells in the kidneys (Fig. 1a) [4]. Interaction with these transporters can cause a non-pathological increase in serum creatinine which has been reported for several drugs such as cimetidine [5], trimethoprim [6], and abemaciclib [7]. An increase in serum creatinine will impact the estimated glomerular filtration rate (eGFR) based on serum creatinine measurements without clinically meaningful alterations in glomerular filtration and renal function. Alternative endogenous markers such as cystatin C are available for assessing GFR and renal function. Cystatin C is freely filtrated by the glomerulus, completely reabsorbed and metabolized by the proximal tubule cells, not secreted by renal transporters like creatinine, and not affected by age, gender, changes in diet, or muscle mass [8, 9].

Evaluation of the interaction between olaparib and renal uptake and efflux transporters in vitro shows inhibition of OCT2, MATE1, and MATE2-K by olaparib at concentrations which can be clinically relevant [10]. Therefore, the reported increase in serum creatinine during olaparib treatment could be a result of inhibition of active tubular secretion (Fig. 1b) and not as a result of reduction in GFR. Since many drugs are excreted by the kidneys and dosed based on the eGFR, including olaparib itself [11], it is important to investigate whether olaparib causes a real decrease in GFR or only an increase in serum creatinine. Dose adjustments of drugs based on the eGFR using creatinine could result in underdosing of patients.

This study aimed to investigate if patients receiving olaparib have an increase in serum creatinine level after start of olaparib treatment and if this increase is reversible after treatment discontinuation. Furthermore, we examined if the induced increase in serum creatinine is likely caused by inhibition of active tubular secretion of creatinine or a reduction in GFR by using cystatin C as an alternative marker for calculating eGFR.

## Methods

### Study design and study population

We retrospectively identified patients using olaparib at the Netherlands Cancer Institute – Antoni van Leeuwenhoek (NKI-AVL) from 2012 until 2020. Institutional review board approval was obtained for this study. Patients with at least one plasma/serum sample available up to 40 days before start of olaparib treatment (baseline) and at least one plasma/serum sample up to 90 days after start of olaparib treatment (during treatment) were included. Patients without a baseline sample but with one sample during treatment and one during (temporary) discontinuation of olaparib treatment (off treatment) were included as well. Samples were considered “off treatment” after at least 3 days of discontinuation of olaparib therapy with an olaparib concentration measured below the lower limit of quantification (LLOQ). Patients received olaparib at several dose levels, according to clinical trial protocols or as prescribed by their treating physician. Plasma/serum samples were collected for therapeutic drug monitoring as part of routine clinical care or for pharmacokinetic assessment and stored at the pharmacy or hospital biobank. We collected data from medical records on age, weight, body mass index (BMI), race, the daily dose of olaparib, tumor type, relevant comedication, creatinine, and c-reactive protein (CRP). Cystatin C levels were measured in the available samples. Renal function was determined by calculating the eGFR using the Creatinine Equation (CKD-EPI 2009) and the Cystatin C Equation (CKD-EPI 2012) as described by Inker et al. [12]. Calculations were performed using R statistical software, version 3.6.0 [13] and the package “nephro” [14].

### Laboratory assessment

Cystatin C was measured in serum or plasma samples at the Department of Laboratory Medicine, NKI-AVL. Analyses were performed using the Tina-quant Cystatin C particle enhanced immune turbidimetric assay with the Cobas 6000 analyzer (Roche, Mannheim, Germany). Latex particles coated with anti-cystatin C antibodies were used and aggregates with cystatin C were turbidimetrically determined at 546 nm. All reagents were ready for use and the calibration range was 0.40–6.80 mg/L. Creatinine was measured routinely on the day of sample collection. Creatinine assays were performed using the Cobas 6000 analyzer with an enzymatic, colorimetric method (Roche, Mannheim, Germany). The calibration range for creatinine was 5–2700 μmol/L. A short validation was performed using plasma and serum samples from 20 patients to compare values of cystatin C and creatinine measured in both matrices.

### Statistical analysis

Statistical analysis was performed using R statistical software, version 3.6.0 [13]. Wilcoxon signed rank tests were used to assess differences in creatinine, cystatin C, and calculated eGFR with both equations at baseline/off treatment and during treatment of olaparib. Multiple linear regression was performed to investigate any association between covariates and change in creatinine and cystatin C levels.

## Results

### Demographics

In total, 66 patients were included of whom at least one plasma/serum sample at baseline/off treatment and one plasma/serum sample during treatment was available. Patient characteristics at baseline/off treatment are presented in Table 1. The majority of patients (65%) were enrolled in a clinical trial and received an olaparib dose of 25 mg once a day (QD), 25 mg BID or 50 mg BID in combination with radiotherapy, with or without cisplatin. A low-dose cisplatin (6 mg/m^2^ on radiotherapy days (5 times a week)) was administrated to 15% of the patients. Sixteen out of 66 patients (23%) were treated with the recommended dose of 300 mg (tablets) or 400 mg (capsules) BID. The 100, 200, and 250 mg BID dose levels were a result of dose reductions due to toxicity or side effects. One patient had an impaired renal function (eGFR based on creatinine) at baseline and, therefore, received an a priori dose reduction to 200 mg BID. CRP, potentially interesting since cystatin C is also an acute phase compound, was not available for most patients. Baseline eGFR values calculated with creatinine and cystatin C were similar.

**Table 1:** Baseline characteristics of all patients

Characteristics		Overall (*n* = 66)
Age (years)	Median	58.5
	Range	29–80
Age group (%)	21–30 years	2 (3.0%)
	31–40 years	8 (12.1%)
	41–50 years	7 (10.6%)
	51–60 years	20 (30.3%)
	61–70 years	17 (25.8%)
	71–80 years	12 (18.2%)
Sex (%)	Male	23 (34.8%)
	Female	43 (65.2%)
Race (%)	White	65 (98.5%)
	Black of African American	1 (1.5%)
Weight (kg)	Median	74.9
	Range	51.3–132.8
BMI (kg/m^2^)	Median	24.9
	Range	19.1–44.9
Creatinine (μmol/L)	Median	72
	Range	48–185
Cystatin C (mg/L)	Median	0.91
	Range	0.49–2.93
eGFR (creatinine) (mL/min/1.73 m^2^)	Mean (SD)	84 (21)
	Range	26–123
eGFR (cystatin C) (mL/min/1.73 m^2^)	Mean (SD)	82 (24)
	Range	18–132
Dose of olaparib (%)	25 mg QD tablets	25 (37.9%)
	*With cisplatin*	*6* (*9.1%*)
	25 mg BID tablets	14 (21.2%)
	*With cisplatin*	*4* (*6.1*)
	50 mg BID tablets	4 6.1%)
	100 mg BID tablets	1 (1.5%)
	200 mg BID tablets	5 (7.6%)
	250 mg BID tablets	1 (1.5%)
	300 mg BID tablets	15 (22.7%)
	400 mg BID capsules	1 (1.5%)
Tumor type (%)	Mamma carcinoma	18 (27.3%)
	Ovarium carcinoma	12 (18.2%)
	Larynx carcinoma	7 (10.6%)
	Oropharynx carcinoma	1 (1.5%)
	NSCLC	28 (42.4%)

*BMI*, body mass index; *eGFR*, estimated glomerular filtration rate; *n*, number of subjects; *SD*, standard deviation; *QD*, once a day; *BID*, twice a day; *NSCLC*, non-small cell lung carcinoma

### Laboratory assessment

We collected 140 samples from 66 patients. Samples at baseline and during olaparib treatment were available from 55 patients. From 15 patients, we collected samples during olaparib treatment and after (temporary) treatment discontinuation. From 4 patients, samples at baseline, during treatment, and after treatment discontinuation were available. The same matrix was available for samples at baseline/off treatment and during treatment, except for 3 patients. During the validation, no statistical differences were found between creatinine or cystatin C values measured in plasma and serum; therefore, both matrices could be used to compare baseline/off treatment samples with during treatment samples. Cystatin C and creatinine concentrations were measured in 140 samples and all concentrations were within the calibration range.

### Effect of olaparib on creatinine, cystatin C, and eGFR

**Fig. 2:**
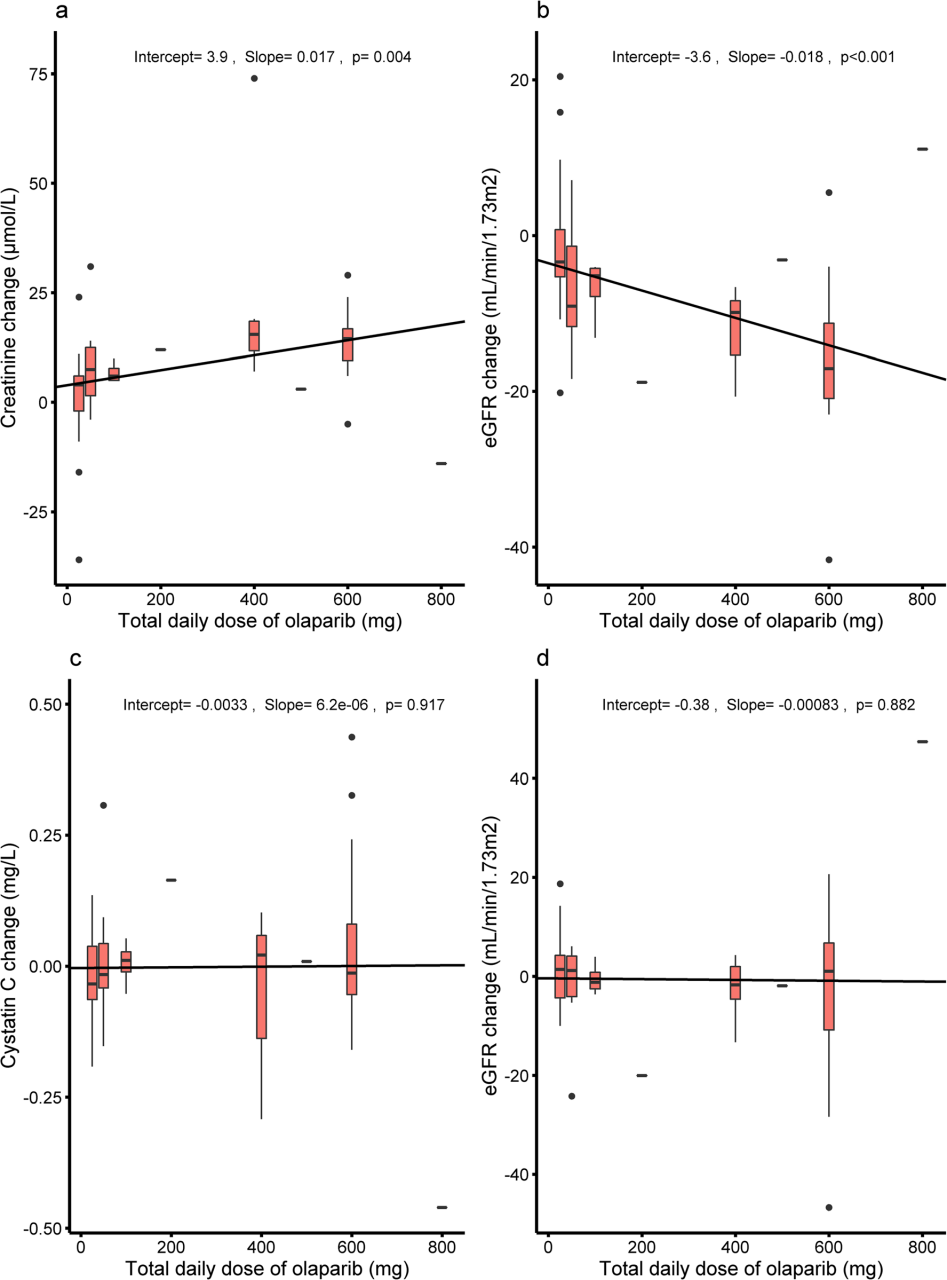
The absolute change in creatinine (**a**), creatinine-derived eGFR (**b**), cystatin C (**c**), and cystatin C–derived eGFR (**d**) stratified by total daily dose of olaparib. Boxplots are given for each dose level where the middle line represents the median with margins representing the 25th and 75th percentiles. A dash is shown in case a single patient represents the dose level. Outliers are represented as dots. Linear regression was performed to calculate the intercept, slope and *p* value

**Fig. 3:**
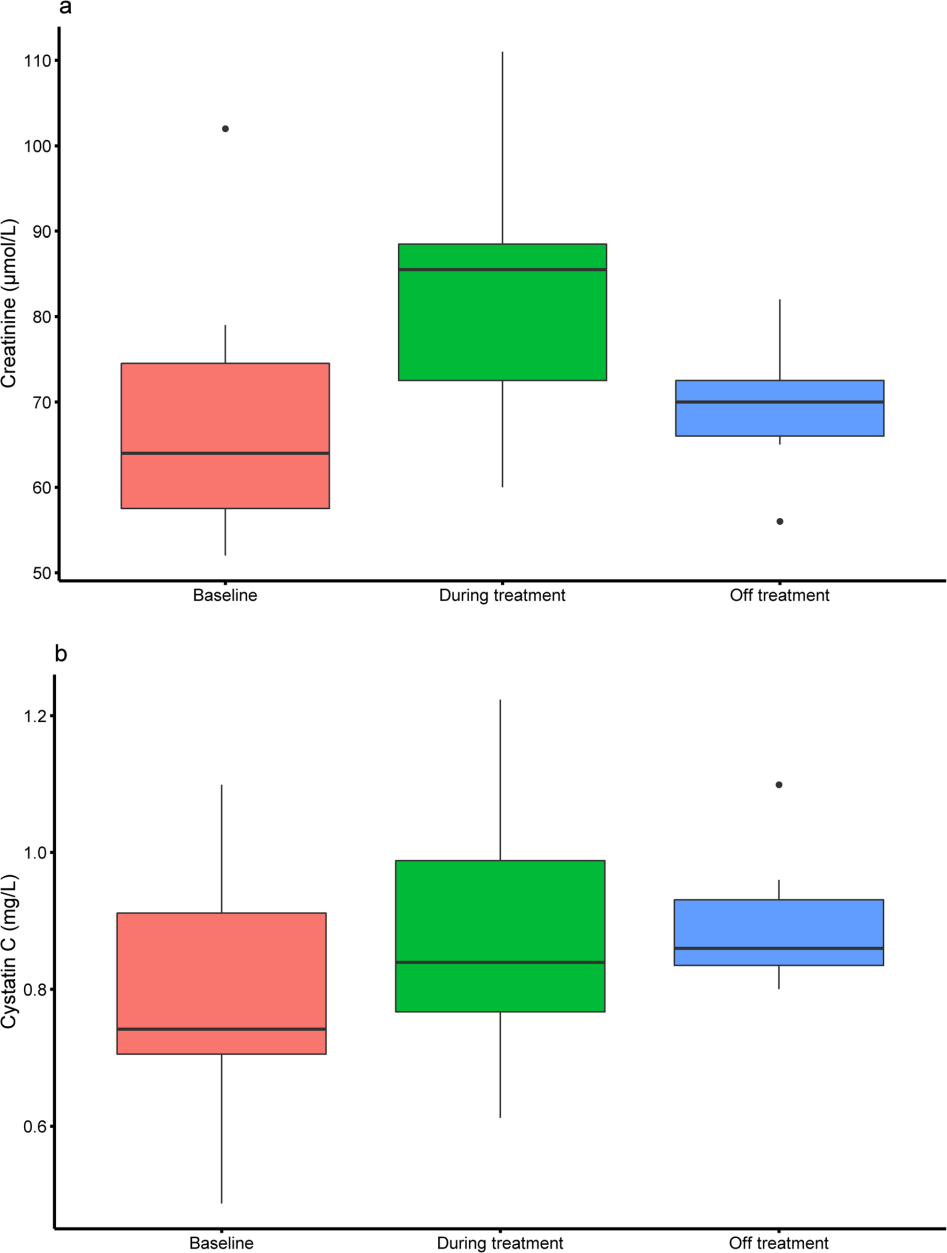
Creatinine (**a**) and cystatin C (**b**) levels of patients treated with olaparib 300 mg BID at baseline (*n* = 11), during treatment (*n* = 18), and after (temporary) treatment discontinuation (*n* = 7). Boxplots are given where the middle line represents the median with margins representing the 25th and 75th percentiles

Overall, olaparib treatment caused a statistically significant increase of 14% in the median creatinine level and decreased the median creatinine-derived eGFR with 13% compared to baseline/off treatment (Table 2). Stratification by dose level and separation of the baseline/off treatment group demonstrates that creatinine and the creatinine-derived eGFR levels at baseline, as well as off treatment, differ significant from levels during treatment (Table 2) in a dose-dependent manner (Table 2 and Fig. 2a and b). A non-significant increase in median creatinine level of 4% was observed at the lowest dose level of 25 mg QD, while higher dose levels show significant increases of 8% at dose level 25 mg BID, 12% at dose level 200 mg BID, and 29% at dose level 300 mg BID. Similarly, a non-significant decrease in median creatinine-derived eGFR of 4% was observed at dose level 25 mg QD, which was significant at dose level 25 mg BID (− 12%), 200 mg BID (− 16%), and 300 mg BID (− 12%). The increase in median creatinine from baseline during olaparib treatment in patients at dose level 300 mg BID (+ 17%) returned back to near baseline after treatment discontinuation (− 23%) (Table 2 and Fig. 3a). Multiple linear regression was performed to test if the dose of olaparib, sex, age, BMI, and concomitant use of cisplatin could predict the change in creatinine and creatinine-derived eGFR. A significant association between dose of olaparib and change in creatinine (*p* = 0.004) and creatinine-derived eGFR (*p* < 0.001) was observed (Fig. 2a and b).

**Table 2:** Creatinine, cystatin C, and eGFR levels of patients at baseline, off treatment, and during treatment of olaparib

Dose level	*N*	Creatinine (μmol/L)					eGFR (mL/min/1.73 m^2^)^a^					Cystatin C (mg/L)					eGFR (mL/min/1.73 m^2^)^b^				
		Baseline	Off treatment	During treatment	Change (%)	*p* value	Baseline	Off treatment	During treatment	Change (%)	*p* value	Baseline	Off treatment	During treatment	Change (%)	*p* value	Baseline	Off treatment	During treatment	Change (%)	*p* value
25 mg QD	25	80 (24)	-	83 (19)	3.8	0.218	89 (22)		86 (25)	− 3.6	0.141	1.01 (0.28)	-	0.98 (0.19)	− 2.7	0.206	76 (32)	-	79 (24)	4.5	0.572
25 mg BID	14	73 (20)	-	79 (26)	8.3	0.009	82 (17)		72 (23)	− 11.9	0.010	0.98 (0.30)	-	0.98 (0.38)	0.2	0.916	78 (29)	-	78 (34)	1.2	0.834
50 mg BID	4	58 (8)	-	66 (11)	13.9	0.098	109 (20)		100 (26)	− 8.7	0.100	0.75 (0.14)	-	0.75 (0.16)	0.0	0.713	108 (15)	-	108 (20)	0.0	0.855
100 mg BID	1	-	64	76	18.8	NA	-	100	82	− 18.4	NA	-	0.75	0.91	22.0	NA	-	107	87	− 18.6	NA
200 mg BID	6	-	81 (28)	93 (33)	11.8	0.036	-	66 (29)	55 (21)	− 16.3	0.036	-	0.92 (0.89)	1.01 (0.73)	9.4	0.787	-	80 (55)	72 (48)	− 9.9	0.418
250 mg BID	1	50	-	53	6.0	NA	121		118	− 2.5	NA	0.82	-	0.83	1.1	NA	104	-	102	− 1.9	NA
300 mg BID	11	64 (17)	-	75 (22)	17.2	0.004	89 (37)		75 (28)	− 15.1	0.004	0.74 (0.21)	-	0.81 (0.25)	9.0	0.541	104 (29)	-	92 (30)	− 11.6	0.541
300 mg BID	7	-	70 (7)	86 (6)	22.9	0.034	-	78 (19)	74 (18)	− 5.0	0.035	-	0.86 (0.10)	0.87 (0.18)	1.2	0.933	-	89 (23)	96 (30)	− 7.9	0.933
300 mg BID combined	18	67 (16)		86 (16)	28.6	< 0.001	85 (35)		75 (24)	− 12.4	< 0.001	0.84 (0.18)		0.84 (0.22)	0.6	0.705	98 (25)		94 (33)	− 4.0	0.670
400 mg BID	1	-	102	88	− 13.7	NA	-	57	68	19.5	NA	-	1.15	0.69	− 40.2	NA	-	64	111	74.3	NA
**Overall**^**c**^	**70**	**72 (22)**		**82 (20)**	**13.9**	**< 0.001**	**86 (26)**		**75 (29)**	**− 13.1**	**< 0.001**	**0.90 (0.30)**		**0.92 (0.29)**	**2.4**	**0.520**	**86 (35)**		**83 (36)**	**− 4.1**	**0.918**

Data are presented as median (IQR). Wilcoxon signed rank tests were performed to calculated *p* values

^a^Calculated with the Creatinine Equation (CKD-EPI 2009)

^b^Calculated with the Cystatin C Equation (CKD-EPI 2012)

^c^66 unique patients. Including 4 patients with one sample at baseline, two samples during treatment, and one sample off treatment

*N*, number of subjects; *QD*, once a day; *BID*, twice a day; *NA*, not applicable

Overall, median cystatin C levels and the median cystatin C–derived eGFR did not differ between baseline/off treatment and during olaparib treatment (Table 2). No significant differences were observed between cystatin C levels at baseline, during olaparib treatment, and after treatment discontinuation in patients at dose level 300 mg BID (Table 2 and Fig. 3b). The dose of olaparib did not relate to a change in cystatin C (*p* = 0.917) and cystatin C–derived eGFR (*p* = 0.882) (Fig. 2c and d).

Two patients showed a substantial change in renal function as measured with cystatin C and cystatin C–derived eGFR. Along with these changes, creatinine and creatinine-derived eGFR were changed as well. One patient at dose level 100 mg BID (change in creatinine, creatinine-derived eGFR, cystatin C, and cystatin C–derived eGFR of + 19%, − 18%, + 22%, and − 19%, respectively) had progression of abdominal metastases and problems urinating at moment of off treatment sample collection. One patient at the 400 mg BID dose level (change in creatinine, creatinine-derived eGFR, cystatin C, and cystatin C–derived eGFR of − 14%, + 20%, − 40%, and + 74%, respectively) was hospitalized at the moment the “off treatment” sample was collected. The patient was suspicious for a bacterial infection and had fever, nausea, was vomiting, and had a CRP of 27 mg/L.

## Discussion

Increases in creatinine levels have been associated with olaparib administration [2]. An increase in creatinine levels can arise from damage to the nephrons resulting in a reduced glomerular filtration rate and reduced creatinine clearance. Another explanation is a decrease in tubular secretion of creatinine caused by inhibition of the OCT2 and/or MATE1/2-K transporters by drugs. In vitro evaluation of olaparib predicted that olaparib inhibits the OCT2 and MATE1/2-K transporters at clinically relevant concentrations. Therefore, we hypothesized that the increases in creatinine are a result of inhibition of tubular secretion and not a reduction in GFR.

Significant increases in creatinine levels (+ 14%) were observed during treatment of olaparib, which is lower than the + 23% reported in literature. However, we included patients at dose levels ranging from 25 mg QD to 400 mg BID. We observed a dose-dependent increase in creatinine levels, with an increase of 29% in patients treated at the recommended dose of olaparib monotherapy (300 mg BID). Paired samples were available between baseline and during treatment and during treatment and off treatment, but not between baseline and off treatment (except for 4 patients). However, creatinine levels at baseline, as well as off treatment, differ statistically significant from creatinine levels during treatment, which suggests the increases in creatinine to be reversible. Since inhibition is reversible, off treatment samples were combined with baseline samples to investigate the overall effect combining all dose levels.

Cystatin C levels were measured to investigate the mechanism behind these increases. Damage to nephrons resulting in a decreased GFR will be reflected in increased cystatin C levels, as well as in increased creatinine levels, both resulting in a decreased eGFR. However, no statistically significant changes in median cystatin C levels and median cystatin C–derived eGFRs were observed. Since cystatin C is not influenced by tubular secretion, it is likely that olaparib does not cause glomerular injury, GFR is not changed following olaparib treatment, and the increase in creatinine is the result of inhibition of renal transporters by olaparib, which is reversible after treatment discontinuation.

Increases in creatinine levels were dose dependent and even observed at dose levels far below the recommended monotherapy dose. Apparently, low doses of olaparib are sufficient to cause inhibition of the renal transporters resulting in an increase in creatinine. The lowest dose level of 25 mg QD had no significant effect on creatinine, while bidaily dosing did have an effect. Creatinine returns back to baseline after discontinuation of a reversible inhibitor of renal transporters [7, 15]. So either the unbound olaparib concentration in blood is too low to cause sufficient inhibition of renal transporters or the once daily dose regimen allows creatinine to return to baseline levels within the dosing interval.

Not only GFR and tubular secretion can impact creatinine levels in blood. Creatinine levels can be affected by diet, malnutrition, and muscle mass, but also by drugs causing kidney damage [3]. Since oncology patients are complex patients using many drugs, comedication was taken into account. The highly nephrotoxic drug cisplatin was used by ten patients during olaparib treatment. Although the administered dose of cisplatin was low, the nephrotoxic effect of cisplatin increases with dose, frequency of administration, and cumulative dose [16] and therefore, cisplatin should be included in the analysis. Linear regression analysis showed no association between cisplatin on change in creatinine. If cisplatin had a significant effect on creatinine due to kidney damage, this would have been reflected in cystatin C levels, which was not the case. Cisplatin is excreted in urine which is mediated by the OCT and MATE transporters. Inhibition of OCT2 prevents accumulation and toxicity of cisplatin, but inhibition of MATE potentiates cisplatin-induced nephrotoxicity. Since olaparib inhibits both transporters, it is not clear if olaparib has protective or inductive effects [4].

While creatinine levels were increased during olaparib treatment in the majority of patients with no change in cystatin C levels, we did observe substantial changes in cystatin C levels along with creatinine levels in two patients. In one of these patients, a substantial difference between cystatin and creatinine-derived eGFR was noted, most likely explained by the inflammation status of this patient. The patient had an elevated CRP concentration of 27 mg/L during olaparib treatment. Inflammation measured by CRP is one of the factors independently positively associated with cystatin C levels and not with creatinine levels. Cystatin C could be a biomarker for inflammation and is also influenced by factors other than renal function [17].

Not only creatinine levels can be increased by olaparib treatment; another important consequence is the potential of drug-drug interactions (DDIs). Inhibition of the OCT2 transporter by olaparib can increase the exposure of drugs transported by the OCT2 transporter, which is, for example, observed for cimetidine co-administrated with metformin [18]. However, it should be investigated further if olaparib causes a clinically relevant increase in exposure of drugs transported by the OCT2 and/or MATE1/2-K transporters.

Our study has some limitations. First, the study had a retrospective design. We were dependent on samples stored in our biobank or pharmacy and baseline samples were not available from all patients. Time of administration of olaparib and time of blood withdrawal were variable in patients not enrolled in clinical trials. Creatinine concentrations can vary and will be maximal after the *t*_max_ of olaparib gradually returning to baseline until the next dose of olaparib is administrated [7]. Second, cystatin C is not a perfect marker for renal function. Cystatin C can be affected by hyperthyroidism, steroid use, and inflammation. Therefore, exogenous markers such as iothalamate, iohexol, or inulin are the most accurate to measure GFR, but these compounds are invasive and expensive. Despite these limitations, the results are significant and convincing. Our study shows that olaparib treatment causes a dose-dependent, reversible increase in creatinine levels and has no effect on cystatin C levels. Therefore, we hypothesize that olaparib does not cause renal injury, has no effect on glomerular filtration, but affects tubular secretion of creatinine resulting in a decreased eGFR. Our observations are in line with a recently published study on the discrepancy between the calculated GFR using creatinine and the measured GFR using GFR scans in patients using PARP inhibitors, which empowers our hypothesis. In this study, a significant decline in creatinine-derived eGFR was found, while the eGFR from renal scans was nearly identical [19]. Here, we confirm these results with an additional and easily accessible biomarker for renal clearance and show the dose dependency of this effect.

Physicians should be aware that in patients taking olaparib, the eGFR based on creatinine can give an underestimation of renal function. This is especially important if the calculated eGFR drops below the level in which dose adjustments of drugs are made. For many drugs, this is below a creatinine clearance of 30 or 50 mL/min, but in some cases even below 80 mL/min. Based on our hypothesis, no dose adjustments are necessary for drugs eliminated by glomerular filtration, including olaparib itself. Dose adjustments made based on the decreased eGFR can result in underdosing of patients. An alternative marker such as cystatin C should be used to accurately assess renal function in patients with moderate to severe renal function.

## Conclusion

This study demonstrates that olaparib treatment leads to a significant, dose-dependent, and reversible increase in creatinine levels. Olaparib likely causes inhibition of renal transporters and does not affect GFR, since the cystatin C–derived eGFR was comparable before/off treatment and during treatment of olaparib. Physicians prescribing olaparib should be aware of the underestimation of renal function using the creatinine-derived eGFR and the potential for drug-drug interactions. It is advised to use an alternative renal marker such as cystatin C to accurately calculate eGFR.

## Data Availability

The datasets generated during and/or analyzed during the current study are available from the corresponding author on reasonable request.
